# From court to the community: improving access to evidence-based treatment for underserved youth involved in the juvenile legal system at-risk for suicide

**DOI:** 10.1186/s12888-023-04824-7

**Published:** 2023-05-05

**Authors:** Jennifer Wolff, Crosby A. Modrowski, Tim Janssen, Hannah E. Frank, Sydney Velotta, Kaitlin Sheerin, Sara Becker, Lauren M. Weinstock, Anthony Spirito, Kathleen A. Kemp

**Affiliations:** 1 Department of Psychiatry & Human Behavior, Brown University Medical School, 1 Hoppin Street, Suite 204, Providence, RI 02903 USA; 2 Center for Alcohol and Addiction Studies, Brown University, 121 S. Main Street, Providence, RI 02903 USA; 3 Bradley Hasbro Children’s Research Center, 1 Hoppin Street, Suite 204, Providence, RI 02903 USA; 4 Center for Dissemination and Implementation Science, Northwestern University Feinberg School of Medicine, 633 N. Clair St, Chicago, IL 60611 USA

**Keywords:** Juvenile legal involved youth, Juvenile legal system, Self-injury, Suicidal thoughts and behaviors, Stepped wedge trial

## Abstract

**Background:**

Juvenile legal involved youth (JLIY) experience disproportionately high rates of suicidal and self-injurious thoughts and behaviors (SSITB). Many JLIY lack access to evidence-based treatment specifically designed to treat SSITB, thereby increasing the overall risk of suicide. The overwhelming majority of JLIY are not placed in secure facilities and almost all incarcerated youth are eventually released to the community. Consequently, SSITB are a major concern of JLIY residing in the community and it is critical that this population has access to evidence-based treatment for SSITB. Unfortunately, most community mental health providers who treat JLIY have not been trained in evidence-based interventions that are specifically designed to SSITB, which often leads to youth experiencing prolonged periods of SSITB. Training community mental health providers who serve JLIY in the detection and treatment of SSITB shows promise for decreasing the overall suicide risk for JLIY.

**Methods:**

The current proposal aims to reduce SSITB among JLIY, and thus reduce mental health disparities in this vulnerable and underserved youth population, by increasing access to evidence-based treatment strategies specifically designed to treat SSITB behaviors. We will implement an agency-wide training among at least 9 distinct community mental health agencies that serve JLIY referred to treatment by a statewide court system in the Northeast. Agencies will be trained in an adapted version of the COping, Problem Solving, Enhancing life, Safety, and Parenting (COPES+) intervention. Training will be implemented via a cluster-randomized stepped wedge trial that proceeds through multiple phases.

**Discussion:**

This research engages multiple systems (i.e., juvenile legal and mental health systems) serving JLIY and has the potential to directly inform treatment practices in juvenile legal and mental health systems. The current protocol has significant public health implications as the primary goals are to reduce SSITB among adolescents involved in the juvenile legal system. By implementing a training protocol with community-based providers to help them learn an evidence-based intervention, this proposal aims to reduce mental health disparities in a marginalized and underserved population.

**Trial registration:**

osf.io/sq9zt

## Introduction

Suicide rates among adolescents have steadily increased and suicide is now the second leading cause of death among 12 to 18-year-olds [1, 2]. Alarmingly, suicide rates have tripled in this age range over the last decade [3] and increased by 62% from 2009 to 2018 alone [4]. In 2018, 2,039 adolescents between the ages of 14 and 18 died by suicide in the United States, which accounted for approximately one-third of injury-related deaths in this age range [4]. Suicide rates are even higher among certain marginalized and underserved populations, including youth involved in the juvenile legal system.

Juvenile legal involved youth (JLIY) represent one group of youth for whom suicidal and self-injurious thoughts and behaviors (SSITB) are disproportionally high. Lifetime rates of suicidal thoughts and behaviors for non-incarcerated JLIY is approximately 14%, with rates even higher in incarcerated youth populations [5, 6]. Between 19 and 29% of JLIY report suicidal ideation in the past 12-months, a rate much higher than adolescents in the general population [7–10]. Rates of non-suicidal self-injury, defined as deliberate destruction of one’s body in the absence of intent to die, are also higher among JLIY than non-JLIY; upwards of 50% of adjudicated youth report previously engaging in non-suicidal self-injury [11]. Suicide attempts, defined as self-harm inflicted with at least *some* intent to die (e.g., self-poisoning, severe cutting, hanging/suffocation, jumping from heights, use of firearm) are also greater among JLIY [12]. For example, between 11 and 15% of JLIY report attempting suicide in the past year compared to 8% of the general adolescent population [7, 9, 10]. More than 80% of the youth arrested each year are not incarcerated and almost all incarcerated youth eventually return to the community [13]. These JLIY living in the community remain at higher risk for SSITB than their non-system-involved peers. Given that SSITB are robust predictors of death by suicide [14], addressing these risk factors may ultimately lower death by suicide in this vulnerable youth population.

Unfortunately, JLIY living in the community who are at risk for suicide encounter many barriers to accessing evidence-based interventions. For example, poor identification of SSITB and lack of streamlined referral procedures within the juvenile legal system are commonly cited barriers to suicidal JLIY receiving mental health care [15]. Additionally, racial, ethnic, and sexual minority youth are disproportionately represented among JLIY [16–18] and are more likely to reside in neighborhoods with limited resources, both of which are associated with decreased access to community mental health treatment [19–21]. Further, those JLIY at risk for suicide who do seek treatment rarely receive evidence-based assessment or treatment for SSITB: this is driven at least in part by the paucity of providers who are familiar with treating JLIY, paired with the fact that providers often work in low-resource community mental health agencies (CMHAs) with limited access to training and resources related to suicide prevention [22]. This is concerning and ultimately increases the overall risk for self-injury, suicide attempts, and death by suicide among JLIY living in the community. The present study aims to address the service gap between those JLIY at risk of suicide and those who receive an evidence-based intervention by testing a multi-component implementation strategy to increase access to evidence-based treatment strategies specifically designed to treat SSITB behaviors in JLIY.

## Rationale for evaluating implementation strategies

To effectively address service gaps in the behavioral health field, it is essential to recognize that the delivery of evidence-based interventions must be complemented by the use of evidence-based implementation strategies. Just as behavioral interventions require specification of core components and careful fidelity monitoring, so do implementation strategies. However, careful monitoring, specification, and evaluation of implementation strategies is relatively rare. The current protocol aims to advance the field by specifying a multi-component implementation strategy and conducting a comprehensive evaluation of its effectiveness.

## Current study/hypotheses

The primary aim of this study is to evaluate a multi-component implementation strategy on the implementation of evidence-based, outpatient treatment strategies to treat SSITB behaviors in JLIY. We will conduct a stepped wedge cluster-randomized trial with nine Rhode Island CMHAs who provide outpatient therapy to JLIY. Consistent with the Exploration, Preparation, Implementation, and Sustainment (EPIS; [23]) phasic approach to implementation, this protocol consists of one comparison phase (Usual Care) and three active phases: Preparation, Implementation, and Sustainment. The evidence-based intervention being implemented is the Juvenile Justice Coping, Problem-solving, Enhancing life, Safety planning, and Parenting (JJ COPES+) [24] treatment protocol, a cognitive-behavioral therapy protocol that has demonstrated effectiveness with JLIY. We hypothesize that: (1) Receipt of the multi-level implementation strategy will increase the number of sessions that use both general cognitive-behavioral therapy strategies and specific JJ COPES + techniques from the Usual Care to the Implementation and Sustainment Phases; (2) Provider fidelity to the JJ COPES + intervention will achieve acceptable ratings on adherence ratings (80% adherent) and competency ratings (> 33 on the JJ COPES Therapy Rating Scale) during the Implementation Phase and scores will remain in this range through the Sustainment Phase; (3) Number of outpatient sessions attended will significantly increase from Usual Care to the Implementation and Sustainment Phases; (4) The prevalence of youth needing emergent psychiatric care due to experiencing SSITB will significantly decrease from the Usual Care to the Implementation and Sustainment Phases.

## Method

### Study design

**Fig. 1:**
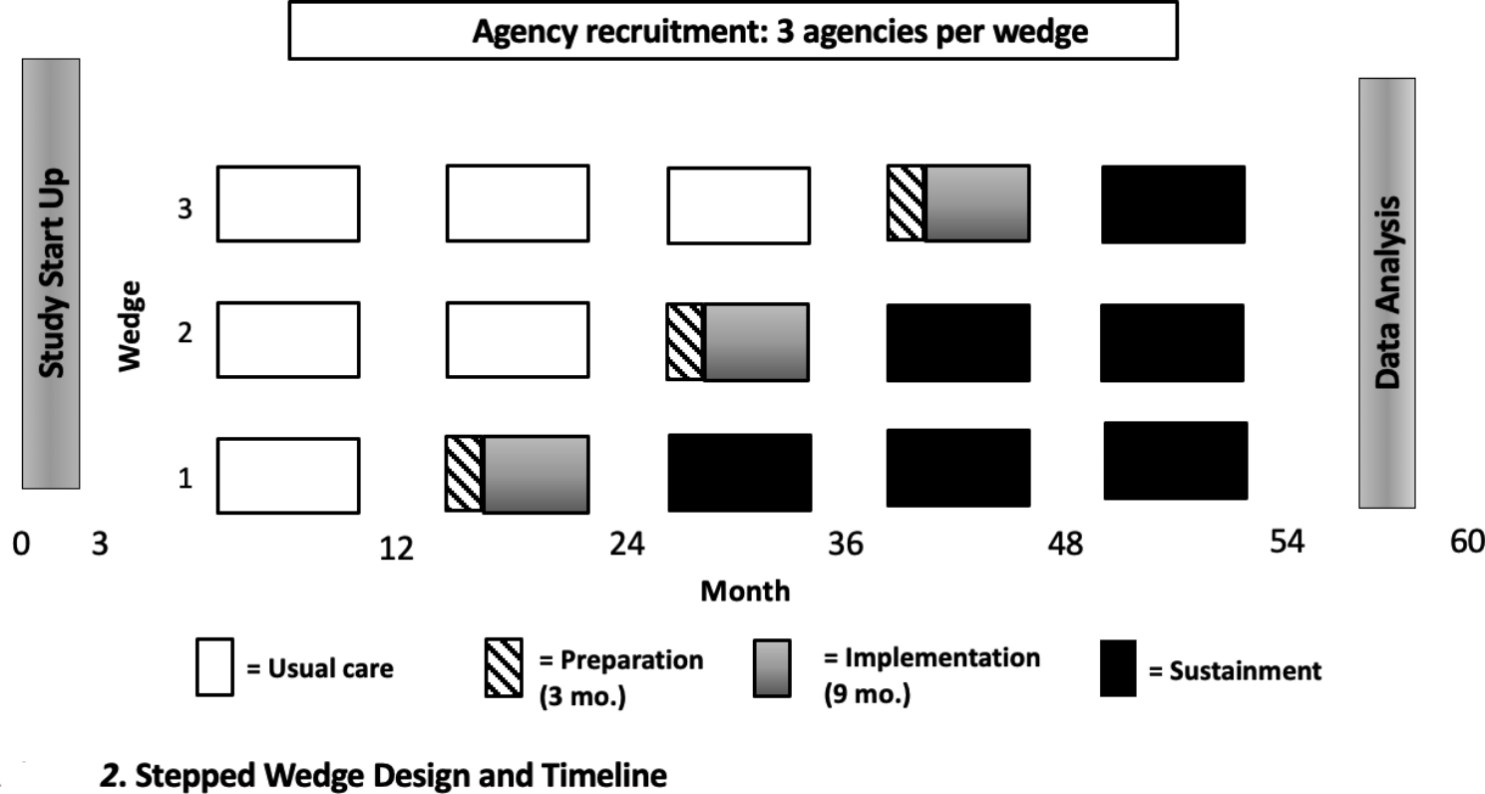
Stepped Wedge Design and Timeline

**Fig. 2:**
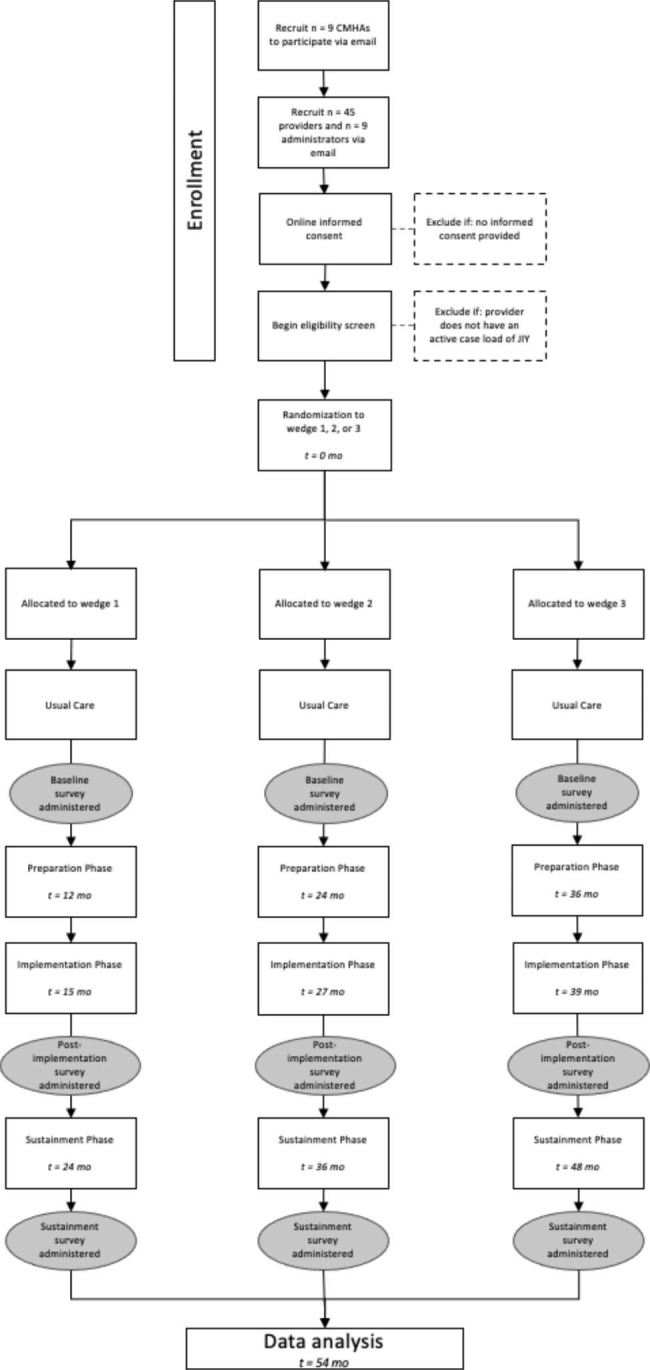
Study Timeline

The current study design is a stepped wedge cluster randomized trial involving nine distinct JLIY-serving CMHAs throughout the state of Rhode Island. The stepped wedge trial has five steps over a 5-year period. This design allows us to sequentially roll out the intervention to all participating programs with each wedge progressing through four distinct phases: Usual Care, Preparation, Implementation, and Sustainment Phases (see Figs. 1 and 2). The stepped wedge design allows all CMHAs to benefit from the multi-component implementation strategy by the end of the study. Thus, the stepped wedge fulfills the dual role of maintaining an unbiased way to determine the order of roll-out while allowing stakeholders to participate knowing their providers and clients will benefit from the investment in collaboration on the research study.

Beginning in month 3 of Year 1 and until each cohort enters the Preparation Phase, we will collect Usual Care data on provider use of evidence-based strategies with their justice-involved clients via electronic medical records. Starting in Year 2, we will randomize one-third of the CMHAs to receive the implementation strategy, followed by another third in Year 3 (see Table 1) and the final third in Year 4. Starting in Year 3, for the first third of CMHAs randomized, we will collect sustainment data from chart reviews of all JLIY served by the providers. Similarly, we will collect sustainment data for the remaining CMHAs following completion of their Implementation phases, which will fall in Year 4 for the second third of CMHAs and Year 5 for the final third of CMHAs, respectively.

**Table 1:** JJ COPES + Intervention Components

JJ COPES + Module	Target	Skill
Safety Planning	Identify warning signs, social support, environmental safety, and reasons to live	Increase emotional awareness, build social supports, and short-term coping skills in crisis
Distress Tolerance/ Emotion Regulation	Identify current ways of coping, and feelings and situations that lead to negative thoughts, feelings, and behaviors	Develop alternative coping strategies to reduce emotional intensity and decrease frequency of suicidal behaviors
Problem Solving	Identify triggers, options, pros/cons, choose solution	Consider safe options and problem solve alternative solutions to effectively manage
Enhancing Life	Identify and set goals behavioral activation, treatment adherence, and supportive behaviors	Set personalized goals for: 1) therapy/medication adherence; 2) exercise; 3) diet; 4) sleep
Parenting	Identify and set goals to improve parenting practices and address barriers	Set goals to improve (1) parent emotion regulation, (2) validation, (3) parental monitoring, and (4) limit setting

### Eligibility criteria

Agencies are eligible to participate if they provide services to JLIY under the age of 19. Agencies must have at least five eligible providers willing to participate. CMHA administrators will inform their staff about the study and explain to providers that they will be contacted by research staff about participation.

Eligible providers must be employed at a participating agency, have active caseloads of JLIY, be willing to be trained in an evidence-based intervention, and participate in implementation efforts. The study aims to enroll a minimum of 45 providers. Participating providers will provide written consent.

Eligible patient charts are those of youth 19 years of age or younger who have a history of justice involvement. For the purpose of this trial, justice involvement is defined as any history of contact with law enforcement or the court system for illegal behavior that resulted in informal (e.g., police diversion) or formal (e.g., court petition) legal involvement. We will extract, on average, five eligible patient medical records from each of the five providers at each of the nine agencies per time point: this corresponds to approximately 225 charts (5 × 5 × 9) per step and 1,125 charts over the course of the study. Of these, 450 charts will be collected during Usual Care, 225 during Implementation, and 450 during Sustainment.

### Intervention

#### **JJ COPES+**

The COping, Problem-solving, Enhancing life, Safety planning, and Parenting (COPES+) [24] treatment protocol covers key evidence-based skills that are integral to treating SSITB in youth. JJ COPES + is derived from an empirically supported CBT manual designed for outpatient treatment of suicidal youth [25, 26]. The JJ COPES + intervention (see Table 1) consists of five skills training modules: Safety Plan, Distress Tolerance/Emotion Regulation, Problem Solving, Enhancing Life, and Parenting. Within each module, there is an emphasis on increasing social support and using behavioral activation. Each session begins by assessing current SSITB and ends with a check-in with caregivers to review ways they can provide support. These skills are typically delivered over the course of 5 sessions lasting about 50 min each. Caregiver involvement includes learning about the JJ COPES + skills, collaboratively developing strategies to support their teen, keeping the home environment safe, and parenting tips. All JJ COPES + materials are available in English and Spanish.

#### **Implementation strategy**

Guided by the EPIS framework, which conceptualizes implementation as a process, implementation activities are divided into one comparison phase (Usual Care) and three active phases. The Exploration Phase was completed in prior formative work with partner CMHAs when designing this protocol. The three active phases are the 3-month Preparation Phase, a 9-month Implementation Phase, and a Sustainment Phase. Across these phases, the key implementation strategies used are provider didactic training, behavioral rehearsals, monthly consultation calls with all staff, and calls with leadership. Specific activities in each of the phases are described in detail in the following sections.

#### **Usual care phase**

During this Phase, agencies will follow usual care practices to serve as the control. Usual care typically includes eclectic therapeutic techniques ranging from supportive therapy to elements of various empirically supported treatments (e.g., cognitive-behavioral therapy). Intake sessions are typically conducted with youth and their caregivers. Follow-up sessions are typically conducted directly with youth and caregivers join sessions at the therapist’s discretion. Depending on which wedge the agency is randomly assigned to, the Usual Care Phase will last between 12 and 33 months.

#### **Preparation phase**

Community mental health agency providers will be trained in the JJ COPES + intervention during months two and three of the Preparation Phase. Training consists of a combination of didactics (provided in-person or via video), demonstrations, and role plays, consistent with best practices for training [27] and takes approximately five hours to complete.

Following the training, staff will be asked to complete a behavioral rehearsal of JJ COPES + skills as such rehearsals have been demonstrated to be consistent with direct observations [28]. The behavioral rehearsals will be randomly selected from the five possible modules. During the rehearsal, a research assistant will play the role of a client and will enact one of a series of prepared clinical vignettes. Multiple vignettes are available to reduce practice effects. The research assistant will be trained in these vignettes to provide consistency across sites. These will be selected from a battery of standardized vignettes that were developed to be representative of common presenting problems and difficulties faced in facilitating the intervention (e.g., patient has difficulty identifying warning signs, there is a gun in the home). Behavioral rehearsal sessions will be audio recorded for coding by research staff.

#### **Implementation phase**

During the 9-month Implementation Phase, all agency providers who receive the training will be asked to complete an additional behavioral rehearsal using the procedures described above to assess fidelity. In addition, all agency providers will be able to participate in a monthly consultation meeting to review the use of the JJ COPES + treatment modules and troubleshoot obstacles. Consultation meetings will occur virtually and will last approximately one hour. Training is limited to these monitoring and consultation sessions to test a feasible procedure for sustained implementation. Monthly consultation calls will be conducted separately with each of the agencies in the Implementation Phase to provide guidance around using JJ COPES + with specific patients.

During Implementation, leadership coaching consultation calls will be held with the CMHA administrator at least twice to address any issues related to staff engagement and training. If staff turnover occurs during Implementation, we will follow the training procedures described below in the Sustainment Phase section for new providers.

#### **Sustainment phase**

Successful sustainability requires that the COPES + treatment strategies be maintained after the expert trainers have withdrawn (i.e., following formal training and consultation with the study team). Immediately following the end of consultation, we will ask trained providers to complete a final behavioral rehearsal with study staff to assess fidelity. All didactic training activities will be video or audio-recorded so administrators can have easily transferrable, low-cost training. We will present a recommended training protocol for replacement providers that consists of watching standardized training videos. In addition, a previously trained provider will conduct simulated role-play sessions of each session with the new provider, as well as a self-rating form. New providers will also be asked to complete self-ratings with JLIY suicidal patients and review with a previously trained provider for 9 months. We recognize that CMHA policies regarding provider training may differ. Consequently, the procedures will be tailored to each site to increase feasibility and acceptability. Our Sustainment Phase will allow us to learn from CMHA administrators and providers about what organizational factors supported the agency to maintain and improve the sustainment of the COPES + model in everyday practice.

### Randomization

Nine agencies will be randomly assigned to one of three wedges, each of which is transitioned to the Preparation Phase at a different time point. Five data collection steps will be included, with Usual Care steps (9–33 months) available for each wedge, followed by the stepped Preparation Phase (3 months) and then the Implementation Phase (9 months). After all sites complete a Usual Care Phase, the study statistician will enter all sites into a randomization spreadsheet to determine the order of training for the 3 wedges of 3 CMHAs. The first wedge of three CMHAs will be monitored for 30 months for Sustainment, the second wedge for 20 months, and the third wedge for 12 months (see Figs. 1 and 2). The sites themselves will not be blind as it is impossible for sites to be blind to randomization while participating in the Implementation Phase.

### Data collection methods

The primary sources of data will be electronic medical records and ratings of therapist fidelity and competence (described below).

### Patient and provider level outcomes

*Electronic medical record data*. Therapy notes will be coded for evidence of provider-level outcomes, including: (1) use of general techniques consistent with cognitive-behavioral therapy (H1) and (2) use of specific JJ COPES + skills (e.g., safety plan), as well as patient-level outcomes, including: (3) treatment attendance (H3); (4) emergency/crisis service use for STB/NSSI (H4); (5) instances of self-injury (H4); and (6) evidence of suicidal thoughts or attempts (H4).

*Therapist Fidelity and Competency*. To evaluate providers’ fidelity and competence in administering the intervention (H2), we will audio record providers’ completion of behavioral rehearsals of COPES + skills with study staff at 3 time points – one each during the Preparation and Implementation Phases, as well as 9 months into Sustainment. Recordings will be rated by research staff for adherence (using a checklist developed for this trial) and competency; 20% will be rated for inter-rater reliability. Competence will be rated with 8 items created specifically for the JJ COPES + intervention and three items from the Cognitive Behavior Therapy Skillfulness Items [29]. A score > 33 will be used as the criterion to indicate competency on the revised JJ COPES + Competency Rating Scale 12-item version. Providers will be paid $50 for participating in the behavioral rehearsals.

### Power analyses and analytic plan

To evaluate power for electronic medical record outcomes, we anticipate extracting approximately 1,125 patient charts throughout the study. For purposes of powering provider-level outcomes, we anticipate extracting data allowing within-subjects comparisons among 6 providers per agency, for 54 providers. Provider turnover is estimated to be approximately 30% among CMHA providers and administrators over the trial. Because chart reviews will be pulled via electronic or paper medical records, it is anticipated that provider turnover will not affect the number of charts reviewed. Monte Carlo power analyses were conducted in Mplus using a two- or three-level mixed-effects latent variable design in a multilevel structural equation modeling (MSEM) design [30] to ensure that the anticipated rate of provider and medical record responses were adequate for robust inferences on outcomes, using 1000 replications.

#### *Power for Hypothesis 1*

For *use of CBT strategies*, we examined a three-level mixed effects model assuming agency-level residual ICC of 3%, and provider-level residual ICC of 49%. Three-level generalized linear mixed effects power analysis suggested > 80% power to detect a 25% fixed change (corresponding to a Cohen’s D of 0.123, i.e. a very small effect) in odds of use of JJ COPES + strategies from Usual Care through Sustainment, using 900 observations, given a base rate of 38% use during Usual Care.

#### *Power for Hypothesis 2*

Hypothesis 2 refers to the implementation goal of achieving 80% adherence and > 33 scoring on the JJ COPES + Competency Rating Scale via administering the intervention. This implementation check confirms that the intervention is sufficiently effective, and as such, is not subject to frequentist testing. Therefore, there are no power analyses appropriate for this Hypothesis.

#### *Power for Hypothesis 3*

We predict that attendance at outpatient care will consist of an average of 4 follow-up appointments during the Usual Care Phase. Similar to the model for determining power for Hypothesis 1, we used a three-level mixed-effects model, with a continuous outcome (number of sessions attended; this outcome would ideally be approximated using a count-based distribution, however, Monte Carlo simulation for three-level models using count-based distributions is not available at this time) and a linear link function for predicting the effect of phase on number of sessions. We assumed a provider-level ICC of 25% and an agency-level ICC of 10%. Since we had no prior data on baseline attendance at outpatient care, we examined the size of the standardized effect we would be powered to detect. We determined that our power to detect a fixed medium effect (Cohen’s *D* = 0.3, B = 0.15) was 98%. The smallest detectable effect was at Cohen’s *D* = 0.22, B = 0.11), for which power to detect was 82%.

#### *Power for Hypothesis 4*

For *Emergency psychiatric care for SSITB*, we assumed minimal agency- (5%) and provider- (5%) level ICC due to the assignment of cases with suicidal ideation to agencies by Rhode Island courts occurring largely at random. Of patients referred by courts, approximately 10% are expected to be at risk for SSITB. Three-level generalized mixed effects power analysis assessing rates of emergency care for SSITB following intervention, assuming a total of 1,125 observations, suggested 85% power to detect a change of 10% before 5% following implementation.

In addition to these analyses, further exploratory analyses will clarify whether provider characteristics, namely demographics (e.g., sex, race, ethnicity), type of professional degree, years of experience, prior training in SSITB interventions, and time in current position, predict the use of CBT/COPES + strategies and treatment outcomes. Exploratory analyses will also examine whether JJ COPES + fidelity ratings in role-plays predict the use of CBT/COPES + strategies and patient level outcomes.

### Institutional review board (IRB) and data safety monitoring board (DSMB)

All methods will be carried out in accordance with relevant guidelines and regulations. This protocol was approved in March of 2022 by the Lifespan IRB. The DSMB for the study is composed of three individuals with expertise in implementation trials for psychological interventions with at-risk youth and a statistical expert. DSMB members do not have any conflicts of interest with the study investigators and are independent from the sponsor. The DSMB meets at least once every 6 months. Adverse events and other unintended effects of the trial will be reported to the IRB, DSMB, and study sponsor.

## Discussion

The purpose of this study is to implement a systems-level intervention to increase access to evidence-based treatment strategies specifically designed to treat SSITB behaviors for JLIY referred to outpatient care. To achieve this aim, we will conduct a stepped wedge cluster-randomized trial with CMHAs who serve JLIY. The significance of the research lies in its ability to advance implementation science within the juvenile legal and mental health systems by testing whether the proposed implementation strategy can improve and sustain fidelity in core evidenced-based treatment strategies (JJ COPES+) for SSITB among JLIY in community mental health care. The clinical significance of this work includes providing information on what factors facilitate the successful implementation and sustainment of evidence-based assessment and treatment for suicidal JLIY living in the community. This research has the potential to advance detection and intervention strategies and yield practice-relevant information regarding scalability, implementation, and dissemination of treatment in the community.

## Data Availability

Data will be available at the end of the study through the NIMH Data Archive.
